# Supplementary material for: Active site specificity profiling datasets of matrix metalloproteinases (MMPs) 1, 2, 3, 7, 8, 9, 12, 13 and 14
Source: Data Brief. 2016 Feb 22;7:299–310. doi: 10.1016/j.dib.2016.02.036 (PMC4777984; doi:10.1016/j.dib.2016.02.036)
Supplement: Supplementary file 10 — Supplementary material [file mmc10.zip › WebPICS_hMMP7_G_1%/P3.html]

 

PICS results


|  |  |
| --- | --- |
| **P3\_A**  32 in 163 sites   19.6 %    effects > 10 perc. pnts.  (vice-versa in brackets)  P2prime\_L: 12.7 (20.4)   P2prime\_V: -11.0 (-15.3) |  |
| **P3\_I**  12 in 163 sites   7.4 %    effects > 10 perc. pnts.  (vice-versa in brackets)  P2\_N: 17.0 (15.7)   P3prime\_A: 41.1 (17.6) |  |
| **P3\_P**  16 in 163 sites   9.8 %    effects > 10 perc. pnts.  (vice-versa in brackets)  P1\_G: 11.4 (15.2)   P1prime\_I: 23.6 (11.4)   P2\_K: 10.2 (11.6)   P3prime\_V: 10.8 (13.3) |  |
| **P3\_V**  24 in 163 sites   14.7 %    effects > 10 perc. pnts.  (vice-versa in brackets)  P1\_K: 10.4 (14.7)   P1\_Q: 11.6 (18.6)   P2prime\_T: 11.0 (16.5) |  |
